# Characterization of thermophilic xylanases from Tengchong Qiaoquan hot spring for lignocellulose bioprocessing and prebiotic production

**DOI:** 10.3389/fmicb.2025.1731615

**Published:** 2026-01-23

**Authors:** Jian-Ling Li, Wei Hu, Xiao-Qi Chen, Lin-Hua Li, Dorji Phurbu, Yan-Yan Zheng, Yi-Wen Zhang, Jing Sun, Zheng-Feng Yang, Kai-Qing Xie, Li-Quan Yang, Yi-Rui Yin

**Affiliations:** 1 College of Agriculture and Biological Science, Dali University, Dali, China; 2 Department of Cardiology, The First Affiliated Hospital of Kunming Medical University, Kunming, China; 3 Xizang Key Laboratory of Plateau Fungi, Institute of Plateau Biology of Xizang Autonomous Region, Lhasa, China; 4 Key Laboratory of Bioinformatics and Computational Biology, Department of Education of Yunnan Province, Dali University, Dali, China; 5 Cangshan Forest Ecosystem Observation and Research Station of Yunnan Province, Dali University, Dali, China; 6 Co-Innovation Center for Cangshan Mountain and Erhai Lake Integrated Protection and Green Development of Yunnan Province, Dali University, Dali, China

**Keywords:** hot spring, lignocellulose bioprocessing, metagenome, prebiotic production, thermophilic xylanases

## Abstract

**Introduction:**

Xylanases are key catalysts for valorizing lignocellulosic biomass, yet many available enzymes lack sufficient thermal stability and exhibit suboptimal activity on complex substrates. To address these limitations, we combined enrichment culturing with metagenomic analysis to discover and characterize two novel GH10 family xylanases, Tc15-Xyn6 and Tc15-Xyn10, from the Qiaoquan geothermal area in Tengchong, Yunnan Province.

**Methods:**

Following molecular cloning, heterologous expression, and purification by Ni^2+^-chelating affinity chromatography, both enzymes were comprehensively profiled.

**Results:**

Tc15-Xyn6 displayed optimal activity at 65 °C and pH 6.6 with a half-life of 2 h at 65 °C, while Tc15-Xyn10 exhibited optimal activity at 60 °C and pH 6.0 with a half-life of 1 h at 60 °C. Both enzymes showed broad pH stability at low temperature: after incubation at 4 °C for 12–24 h across pH 4.0–10.0, Tc15-Xyn6 and Tc15-Xyn10 retained more than 60 and 40% of their initial activity, respectively. Both efficiently hydrolyzed xylan in alkali-treated wheat straw, rice straw, and corn stover, as well as xylan from hot water–treated wheat bran, but yielded distinct product profiles: Tc15-Xyn6 primarily produced xylobiose and xylotetraose, whereas Tc15-Xyn10 generated xylotriose as the main product. The resulting xylooligosaccharides significantly promoted the growth of *Lactococcus lactis*. Kinetic analyses showed *K*_m_ and *V*_max_ values of 4.675 mg/mL and 125 μmol/min/mg for Tc15-Xyn6, and 9.36 mg/mL and 59.52 μmol/min/mg for Tc15-Xyn10.

**Discussion:**

Collectively, Tc15-Xyn6 and Tc15-Xyn10 combine thermophilicity, thermostability, near-neutral pH preference, and strong performance on complex lignocellulosic substrates, supporting their application in feed processing and targeted production of prebiotic xylooligosaccharides from biomass.

## Introduction

Xylan, a major component of plant cell wall hemicellulose, consists of a β-1,4-linked xylose backbone that is frequently substituted with acetyl groups, arabinose, glucuronic acid, and other side chains, resulting in substantial structural diversity and complexity (Li et al., 2022; Paschoa et al., 2024; Cheng et al., 2025; Andressa et al., 2022). Xylanases (EC 3.2.1.8) specifically hydrolyze this backbone to produce xylooligosaccharides (XOS) of varying degrees of polymerization (DP), and play a central role in fiber degradation and lignocellulose valorization. Based on sequence and domain characteristics, xylanases are classified into multiple glycoside hydrolase (GH) families, with GH10 and GH11 being the most prevalent and widely distributed (Li et al., 2022; Nguyen et al., 2018). Glycoside hydrolase family 10 (GH10) xylanases typically exhibit diverse substrate specificities and possess a canonical (*α*/*β*)8 barrel fold (TIM barrel), with most containing a carbohydrate-binding module (CBM) (Collins et al., 2005; Wu X. et al., 2022; Wu Z. et al., 2022). In contrast, most glycoside hydrolase family 11 (GH11) xylanases only have a catalytic domain and act exclusively on d-xylose-containing substrates, usually adopting a β-sheet structure (Paes et al., 2012; Wu X. et al., 2022; Wu Z. et al., 2022). Traditional pure-culture techniques capture less than 1% of environmental prokaryotes under laboratory conditions, severely hindering discovery of novel functional enzymes, especially thermophilic and thermostable xylanases (Uchiyama and Miyazaki, 2009). Metagenomics circumvents cultivation bottlenecks by directly accessing microbial community DNA from environmental samples, enabling high-throughput discovery of functional genes and biocatalysts (Tansirichaiya et al., 2023). Extreme environments such as hot springs often harbor enzymes with exceptional thermal stability and environmental tolerance, making them prime habitats for discovering thermostable xylanases.

Thermostable xylanases hold significant biotechnological value in industries such as food, feed, biofuels, textiles, and pulp and paper (Dahiya et al., 2024; Kumar S. et al., 2016; Kumar V. et al., 2016). However, natural xylanases typically exhibit optimal activity between 40 and 60 °C with poor thermal stability, failing to meet the high-temperature requirements of industrial processes. Their widespread industrial application is further constrained by insufficient thermal stability, limited catalytic efficiency toward complex substrates, and high production costs (Jiang et al., 2020). Currently, numerous industrial operations necessitate high-temperature conditions. For instance, in the feed industry, phytase and xylanase must undergo conditioning at 74 °C or 85 °C for 30 s prior to granulation (Evans et al., 2021). In the pulp and paper industry, xylanases are employed in pulp bleaching—a process commonly conducted at elevated temperatures ranging from 65 to 75 °C (Beg et al., 2001). In the biofuel industry, xylanases play a pivotal role and are required to withstand high-temperature and high-pressure environments (Saha et al., 2003). Combining biomass pretreatment with thermostable enzyme degradation is currently an important approach for the efficient conversion of biomass. Zhou H. et al. (2024) and Zhou J. et al. (2024) reported enhanced ethanol yields from waste paper through hydrothermal pretreatment combined with enzymatic hydrolysis, while Ma et al. (2022) demonstrated that wheat straw pretreated with deep eutectic solvents (DES) for 6 h achieved a digestion rate of 89.98% when hydrolyzed enzymatically at 150 °C, representing a significant improvement compared to the 39.97% digestion rate observed with 12 h pretreatment at 90 °C. These studies not only demonstrate the important role of optimized pretreatment strategies in overcoming lignocellulose recalcitrance but also emphasize the need for thermostable and stable enzyme preparations under high-temperature conditions. Therefore, in the conversion of lignocellulosic biomass (e.g., agricultural waste), besides effective pretreatment strategies, thermostable xylanases are indispensable for enhancing hydrolysis efficiency and process robustness (Dhiman et al., 2008; Sarangi and Thatoi, 2024).

In the feed industry, besides their application in feed granulation, xylanases are particularly valuable when added as exogenous enzymes to feed to promote nutrient absorption. Statistics from the Food and Agriculture Organization (Canton, 2021) indicate that approximately 60% of cereal raw materials (e.g., wheat, corn, and barley) in compound feeds are rich in non-starch polysaccharides (NSPs), predominantly composed of structural carbohydrates such as xylan. In feed production, cereal raw materials rich in NSPs account for a relatively high proportion, highlighting the urgent demand for efficient NSP-degrading technologies. Xylanase-based bioprocessing is an effective solution to address this challenge. NSPs are difficult to degrade by endogenous enzymes in monogastric animals, easily increasing the viscosity of intestinal contents, inhibiting nutrient digestion and absorption, and inducing diarrhea and growth retardation (Annison and Choct, 1991; Bedford, 2018). The addition of exogenous enzymes to diets has become a conventional strategy to improve feed utilization efficiency. Among these, xylanases can significantly enhance the utilization of wheat-based diets, reduce chyme viscosity, and optimize intestinal physiology (Arczewska-Wlosek et al., 2019). In pigs, the synergy between xylanases and β-glucanases can reduce jejunal viscosity and immune stress, improve intestinal structure and nutrient digestibility, thereby enhancing growth performance (Choi et al., 2024). In aquatic diets, the addition of NSP-degrading enzymes can increase specific growth rate, feed efficiency, and nitrogen retention, while reducing ammonia nitrogen emissions (Sinha et al., 2011). In ruminants, exogenous xylanases can further enhance rumen fermentation efficiency through feed pretreatment (Beauchemin et al., 2003).

Xylooligosaccharides (XOS) produced by xylanase hydrolysis exhibit prebiotic properties, promoting the proliferation of beneficial bacteria and improving intestinal health. As a result, they serve as crucial raw materials for developing functional feeds, foods, and healthcare products (Mu et al., 2024; Cruz-Guerrero et al., 2022). Diabetes mellitus is a severe chronic endocrine/metabolic disorder that can lead to various life-threatening complications. Studies have shown that the gut microbiota can modulate intestinal barrier function and host immunity, thereby regulating glucose and lipid metabolism processes. Consequently, the gut microbiota is closely associated with the development and progression of diabetes and has emerged as a potential target for diabetes treatment (Su et al., 2023). Therefore, screening novel xylanases with neutral to slightly acidic optimal pH, thermostable (or thermophilic) characteristics, and tolerance to various industrial processing conditions is a critical direction for industrial applications.

In this study, we combined enrichment culture with metagenomics to discover two novel GH10 xylanase genes (Tc15-Xyn6 and Tc15-Xyn10) from the Qiaoquan hot spring in Tengchong, Yunnan province, China. These genes were heterologously expressed in *Escherichia coli*, and the purified enzymes were systematically characterized for temperature and pH optima and stability, substrate specificity, and kinetics. We further evaluated their hydrolytic performance on pretreated agricultural lignocellulosic substrates and assessed the prebiotic potential of the resulting XOS. This work provides new enzymatic resources with near-neutral pH preference, thermophilicity, and thermostability, together with strong performance on complex substrates, to support feed processing and cost-effective production of prebiotic XOS from lignocellulosic biomass.

## Materials and methods

### Sample collection and metagenomic sequencing

Sediment samples were collected from the Qiaoquan hot spring in the Tengchong Rehai Geothermal Area, Yunnan Province (24°56′38.4″N, 98°26′24″E). Samples underwent enrichment culture for 1 month using filter paper as the sole carbon source. Total DNA was extracted using the MOBIO DNeasy PowerSoil Kit (United States) according to the manufacturer’s instructions. Metagenomic sequencing was performed by GENEWIZ (Suzhou, China) on the Illumina HiSeq 2,500 platform. *De novo* assembly was conducted with Velvet v1.2.08 (Zerbino and Birney, 2008). Assembled contigs were preliminarily annotated on the JGI IMG/M ER platform^1^. Functional annotation of genes and open reading frames (ORFs), and domain identification, were performed using the COG (Tatusov, 2001), KEGG (Nakaya et al., 2012), and Pfam (Finn et al., 2007) databases.

### Sequence screening and bioinformatics analysis of Tc15-Xyn6 and Tc15-Xyn10

Candidate xylanase genes were identified based on KEGG and COG annotations integrated with Pfam domain information, yielding two novel GH10 family xylanases, Tc15-Xyn6 and Tc15-Xyn10. Their coding sequences were optimized for *Escherichia coli* codon usage for subsequent cloning and expression. Gene and protein sequence homology was assessed using BLASTx and BLASTp, respectively^2^. Signal peptides were predicted with SignalP^3^. Physicochemical properties were evaluated using ExPASy ProtParam. Closely related sequences from the same or neighboring genera were selected as reference sequences via NCBI BLASTp, and sequences from more distant genera were included as outgroups. Multiple sequence alignments were performed in ClustalX (Thompson et al., 1997), and a phylogenetic tree was constructed in MEGA7 using the Maximum Likelihood method with the Poisson correction model (Kumar S. et al., 2016; Kumar V. et al., 2016). Alignments were visualized with ESPript (Larkin et al., 2007), and three-dimensional homology models were built using SWISS-MODEL. Structural visualization and analysis were conducted in PyMOL.

### Gene amplification and construction of recombinant expression vectors

The DNA and protein sequences of tc15-xyn6 (Accession No.: PX522158) and tc15-xyn10 (Accession No.: PX522161) have been deposited in the NCBI GenBank database. For seamless cloning, 5′ primer extensions homologous to the pSHY211 vector ends (linearized with EcoRI and HindIII) were added to each primer. The primers were: Tc15-Xyn6-F (CATCATCATCATCATCATGAA CTGGCGCTGTGTCTGCTCTCCT), Tc15-Xyn6-R (GTGCTCGAGTGCGGCCGCAAG CTCGAGCCGCACACGAACTAC), and Tc15-Xyn10-F (CATCATCATCATCATCATGAA ATGGCCCTGGCCCAGTCTG), Tc15-Xyn10-R (GTGCTCGAGTGCGGCCGCAAG GGGTCGTTGCAAGGCTTGCT). The underlined part of the sequence is the homologous sequence of the restriction endonuclease digestion vector. The underlined 5′ segments (not shown here) are homologous to the EcoRI/HindIII-linearized pSHY211 ends. The PCR program was: 95 °C for 180 s; 10 cycles of 98 °C for 20 s and 68 °C for 150 s; 30 cycles of 98 °C for 20 s, 55 °C for 30 s, and 72 °C for 150 s; final extension at 72 °C for 10 min. PCR fragments were assembled into pSHY211 using the pEASY-Uni Seamless Cloning and Assembly Kit (TransGen Biotech, China) to generate pSHY211-Tc15-Xyn6 and pSHY211-Tc15-Xyn10. Recombinant plasmids were transformed into *E. coli DH5α* for cloning and expression. Transformants were selected on LB agar containing kanamycin (50 μg/mL). Plasmid DNA was purified using a commercial kit (Sangon Biotech, China) and sequence-verified.

### Heterologous expression and purification of recombinant proteins

A single *E. coli DH5α* colony harboring the confirmed expression plasmid was inoculated into 100 mL LB medium containing 50 μg/mL kanamycin and incubated at 37 °C, 180 rpm for approximately 7 h. The temperature was then reduced to 25 °C, and incubation continued at 180 rpm for an additional 12 h to promote soluble expression. Cells were harvested by centrifugation at 4,000 × g for 20 min at 4 °C and resuspended in lysis buffer (prepared according to the manufacturer’s instructions or established protocols). Cells were disrupted by sonication on ice, and the lysate was clarified by centrifugation at 12,000 × *g* for 15 min at 4 °C to obtain the crude enzyme supernatant.

His-tagged recombinant proteins were purified using a Ni^2+^-chelating affinity column (TransGen Biotech, China) following the manufacturer’s protocol and as described by Yin et al. (2017). Protein concentration was determined by the Bradford assay (Sangon Biotech, Cat. No. C503031) using bovine serum albumin as the standard. Purity and apparent molecular mass were assessed by 12% SDS-PAGE.

### Xylanase activity assay

Xylanase activity of Tc15-Xyn6 and Tc15-Xyn10 was measured following Yin et al. (2023). Reducing sugars were quantified by the 3,5-dinitrosalicylic acid (DNS) method with xylose as the standard (Miller, 1959). Unless otherwise specified, reactions contained 1% (w/v) beechwood xylan in appropriate buffer and were incubated at the enzyme’s optimal temperature under initial-rate conditions. After adding DNS reagent, mixtures were boiled for 5 min, cooled to room temperature, and absorbance was measured at 540 nm. Reducing sugar concentrations were calculated from a xylose calibration curve. One unit (U) of xylanase activity was defined as the amount of enzyme releasing 1 μmol of xylose equivalents per minute under standard assay conditions. Each assay included a heat-inactivated enzyme control and was performed with three biological replicates.

### Enzymatic characterization

Optimal temperature and thermostability: Relative activity to temperature was measured at pH 7.0 over 20–80 °C to determine the temperature optimum. Thermostability was evaluated by preincubating enzymes at the indicated temperatures for 0, 20, 40, 60, 80, 100, and 120 min, followed by measurement of residual activity under standard conditions.

Optimal pH and pH Stability: Relative activity in response to pH was determined using two buffer systems tailored to their effective pH ranges: sodium citrate–dibasic sodium phosphate buffer (pH 3.0–8.0) and glycine-NaOH buffer (pH 8.0–11.0). For pH stability analysis, purified enzymes were incubated at 4 °Cfor 12 h and 24 h in the aforementioned buffers spanning pH 3.0–11.0 (i.e., sodium citrate-dibasic sodium phosphate buffer for pH 3.0–8.0 and glycine–NaOH buffer for pH 8.0–11.0). Residual enzyme activity was then measured under standard assay conditions.

Effects of metal ions and chemical reagents: Metal ions (K^+^, Mg^2+^, Fe^3+^, Ca^2+^, Zn^2+^, Co^2+^, Cu^2+^, Ag^+^, Mn^2+^, Pb^2+^, Ni^2+^, Ba^2+^, Cd^2+^, Al^3+^) were added individually at final concentrations of 1 mM and 10 mM. Chemical reagents (EDTA, PMSF, Tween 80, methanol, ethanol, SDS, urea, β-mercaptoethanol, CTAB, DTT, Triton X-100, and isopropanol) were added at 0.1 and 1% (w/v or v/v, as appropriate). Relative activities were determined under standard assay conditions, with reactions lacking added ions or reagents serving as controls.

Substrate specificity and kinetic parameters: Relative activity was assessed using 1% (w/v) substrates: beechwood xylan, corn cob xylan, sugarcane bagasse xylan, carboxymethylcellulose sodium (CMC-Na), Avicel, and cellobiose, under each enzyme’s optimal temperature and pH. For kinetics, substrate concentrations ranged from 0.1 to 20 mg/mL under optimal conditions. Michaelis–Menten parameters (*K*_m_ and *V*_max_) were calculated from Lineweaver–Burk double-reciprocal plots.

### Thin-layer chromatography (TLC) analysis of hydrolysis products

To profile hydrolysis products, 1% (w/v) beechwood xylan was incubated with 10 μg of purified enzyme (Tc15-Xyn6 or Tc15-Xyn10) for 12 h under the respective optimal conditions. Aliquots were spotted onto Silica gel 60 plates (Merck, Darmstadt, Germany). Chromatography was performed with n-butanol/acetic acid/water (2:1:1, v/v/v) as the mobile phase. Plates were sprayed with 5% (v/v) H_2_SO_4_ in ethanol and baked at 120 °C for 10 min to visualize sugars. Xylose (X1), xylobiose (X2), xylotriose (X3), and xylotetraose (X4) served as standards.

### Preparation of xylan substrates and enzymatic hydrolysis

Raw material pretreatment and xylan extraction: Sugarcane bagasse, wheat bran, wheat straw, poplar wood residue, corn stover, corn cob, rice straw, and pine wood residue were washed thoroughly, air-dried overnight, ground, and sieved to 2–10 mm. For each material, 50 g was extracted in 1.5 L of 2% (w/v) NaOH at 80 °C for 90 min. After cooling, the slurry was filtered through cotton cloth. The filtrate was adjusted to pH 5.0 with glacial acetic acid, and 95% (v/v) ethanol was added to a final concentration of 50% (v/v) to precipitate xylan. After standing overnight, the precipitate was collected by centrifugation, washed with distilled water, and dried at 50 °C to constant weight to obtain alkali-extracted xylan (Faryar et al., 2015). For high-temperature water-treated substrates, NaOH was replaced with an equal volume of water; remaining steps were unchanged.

Enzymatic hydrolysis: For each substrate, 0.2 g xylan was incubated with either 80 μg purified Tc15-Xyn6 (enzyme solution 108.69 μg/mL) or 80 μg purified Tc15-Xyn10 (enzyme solution 357.14 μg/mL) in a total volume of 5 mL pH 6.6 buffer. Reactions were mixed thoroughly and incubated at 55 °C. Samples were collected every 2 h up to 12 h, and then every 12 h up to 48 h. Supernatants were obtained by centrifugation at 10,000 rpm for 3 min, and reducing sugars were quantified by the DNS method. For each substrate, a no-enzyme blank was included. All experiments were performed with at least three independent biological replicates.

### Impact of prebiotic products on lactic acid bacteria growth and co-culture experiments

Prebiotic product preparation and monoculture evaluation: Enzymatic hydrolysates generated in Section 2.8 were used as prebiotic products. An equal volume of hydrolysate was added to 10 mL LB medium, followed by inoculation with 200 μL of a *lactic acid bacterium* seed culture (e.g., *Lactococcus lactis*). LB medium without prebiotic served as the control. Cultures were incubated at 37 °C with shaking. OD600 was recorded every 2 h up to 12 h, and then every 12 h up to 72 h to construct growth curves. All assays were performed in triplicate.

Simulated gut co-culture: Equal inocula of EGFP-labeled *E. coli* and *lactic acid bacteria* were co-cultured in LB medium with or without the prebiotic product. The experimental group contained prebiotic; the negative control contained no prebiotic. Cultures were incubated at 37 °C with shaking. OD600 and fluorescence intensity (EGFP, excitation 485 nm) were measured at the same time points as above. Each condition was performed in triplicate to assess the impact of the prebiotic on competitive dynamics and proliferation of *lactic acid bacteria* versus other community members.

### Molecular docking

The Tc15-Xyn6 protein was used as the receptor, with small-molecule xylooligosaccharides (XOS) X2 and X4 as docking ligands. Simultaneously, Tc15-Xyn10 protein served as the receptor, with X3 as the docking ligand for molecular docking assays. The three-dimensional (3D) structures of Tc15-Xyn6 and Tc15-Xyn10 were constructed using SWISS-MODEL, and small-molecule XOS (X2, X3, and X4) were utilized as docking ligands (Yang and Han, 2018). Enzyme-ligand docking simulations were performed using the AutoDock Vina 1.5.6 platform. The docking grid parameters applied for all XOS are provided in the Supplementary material. This grid box encompassed the entire active site cleft, with the +1 and −1 subsites located at its center. Ligand atoms possessed high degrees of freedom; during ligand parameterization, active torsion selection and torsion root identification were conducted. In this process, the rotatable and non-rotatable bonds present in the substrate molecules were evaluated. Based on the sequence identity and query coverage of the protein complexes, the ligand conformation most compatible with the protein complex was selected. The binding energy scores of the obtained docking structures were ranked. The Protein-Ligand Interaction Profiler (PLIP) was utilized to optimize the active site residues. The criterion for determining hydrogen bonds was that the maximum distance between donor and acceptor atoms should be less than 3.4 Å. To confirm the interactions between the enzyme and substrate, visual analysis was performed using PyMOL.

### Statistical analysis

Unless otherwise specified, all experiments were conducted with three biological replicates, and mean values were used for analysis. Statistical analyses were performed in SPSS 20.0. Data are reported as mean ± standard error of the mean (SEM). One-way ANOVA was used, followed by Tukey’s *post hoc* test for multiple comparisons. In all cases, *p* < 0.05 was considered statistically significant.

## Results

### Cloning and sequence analysis of xylanase genes

**Figure 1:**
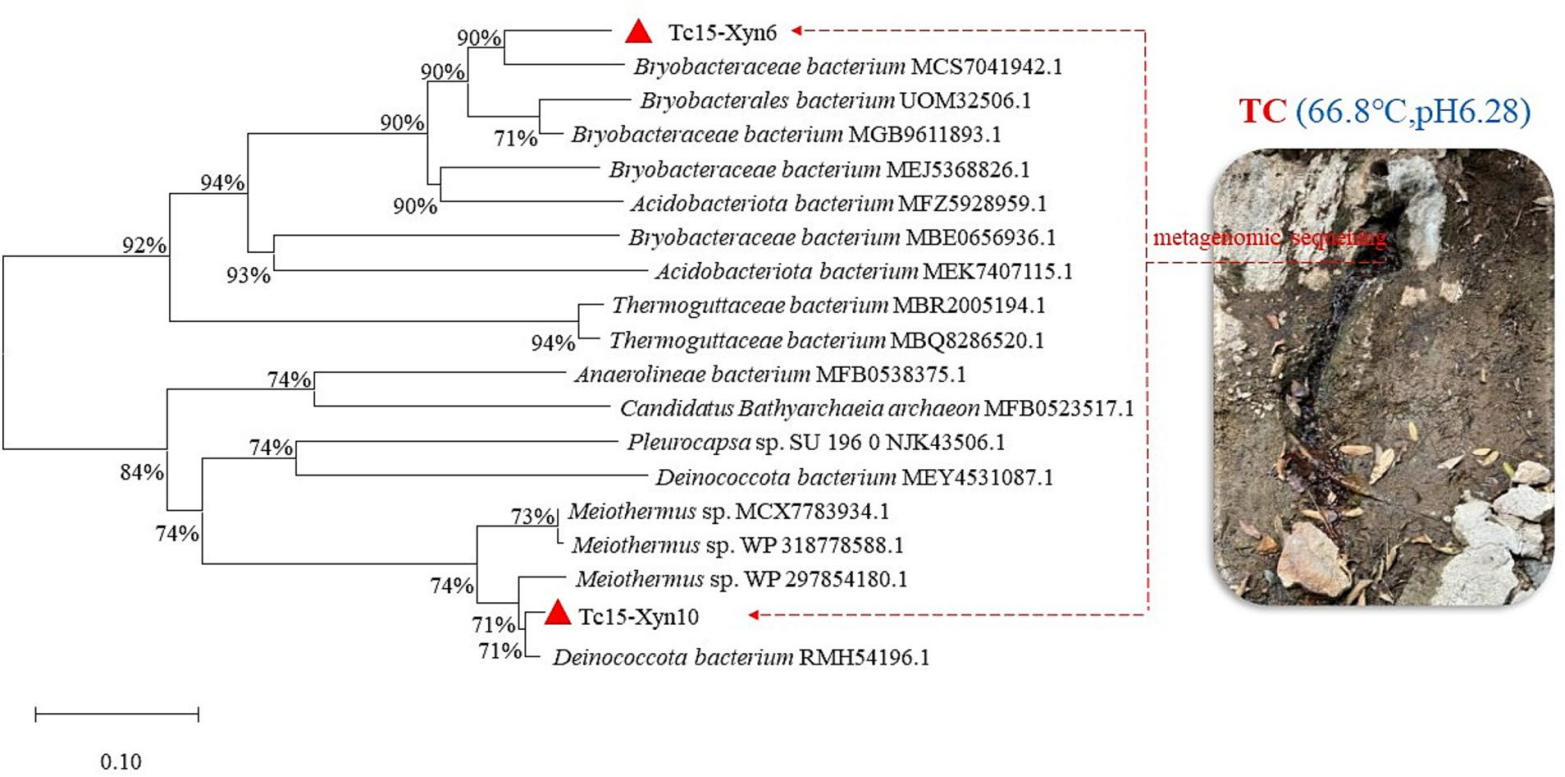
Phylogenetic tree based on amino acid sequence homology of Tc15-Xyn6 and Tc15-Xyn10.

**Figure 2:**
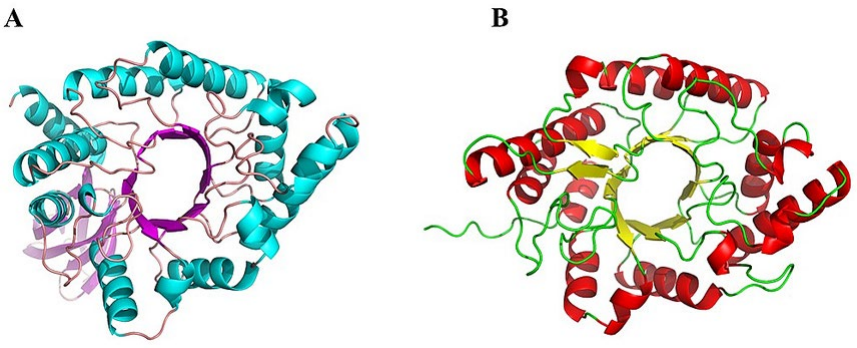
Homology models of Tc15-Xyn6 **(A)** and Tc15-Xyn10 **(B)**, showing the GH10 TIM-barrel topology.

Metagenomic sequencing and mining of enriched cultures and total DNA from Qiaoquan hot spring sediments led to the identification of two putative xylanase genes, Tc15-Xyn6 and Tc15-Xyn10. Tc15-Xyn6 is 1,212 bp in length and encodes a 400-amino-acid protein. A signal peptide was predicted at residues 1–21, and the GH10 catalytic domain was mapped to residues 104–354. Tc15-Xyn10 is 1,305 bp and encodes a 315-amino-acid protein with a GH10 domain spanning residues 53–315; SignalP did not detect a typical N-terminal signal peptide. Theoretical molecular masses were 44.64 kDa (Tc15-Xyn6) and 35.84 kDa (Tc15-Xyn10), with predicted pI values of 5.96 and 7.77, respectively. BLAST and phylogenetic analyses showed that Tc15-Xyn6 clusters with a Bryobacteraceae bacterium xylanase (accession MCS7041942.1; 87.37% identity), while Tc15-Xyn10 clusters with a Deinococcota bacterium xylanase (accession RMH54196.1; 99.37% identity) (Figure 1). Homology modeling indicated that both proteins adopt the canonical (*α*/*β*)8 TIM-barrel fold characteristic of GH10 enzymes (Figure 2).

### Heterologous expression and protein purification

**Figure 3:**
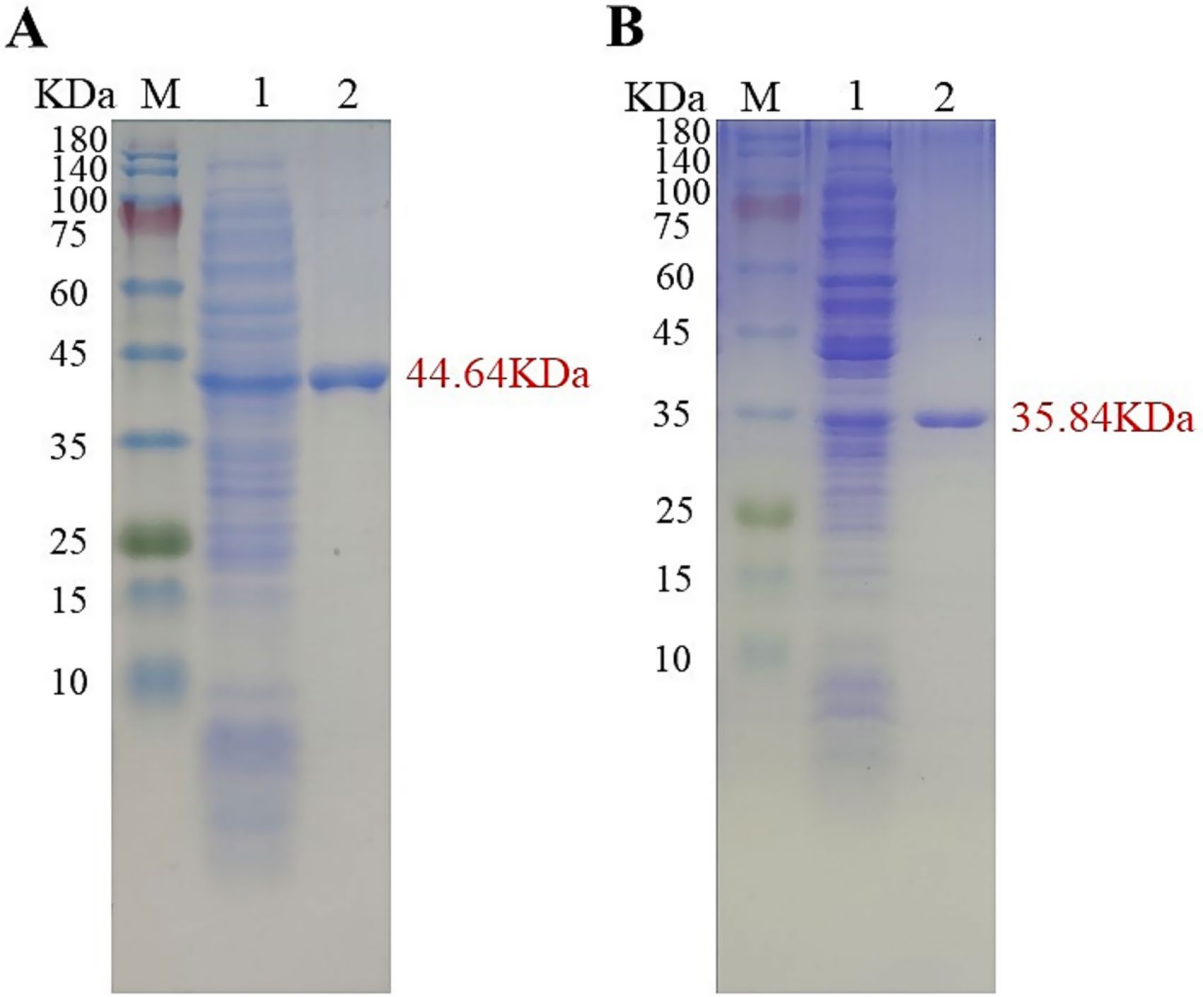
SDS-PAGE analysis of recombinant xylanases. **(A)** *Tc*15-Xyn6; **(B)** *Tc*15-Xyn10. Lane M, protein marker; lane 1, cell lysate; lane 2, purified protein.

Tc15-Xyn6 and Tc15-Xyn10 were cloned into pSHY211 as His-tag fusions and verified by sequencing. Following Ni^2+^-NTA affinity purification, 12% SDS-PAGE revealed single bands at approximately 44.64 kDa (Tc15-Xyn6) and 35.84 kDa (Tc15-Xyn10), consistent with theoretical masses and indicating successful expression and purification (Figure 3).

### Effects of temperature and pH on recombinant xylanase activity and stability

#### Temperature optima and thermostability

**Figure 4:**
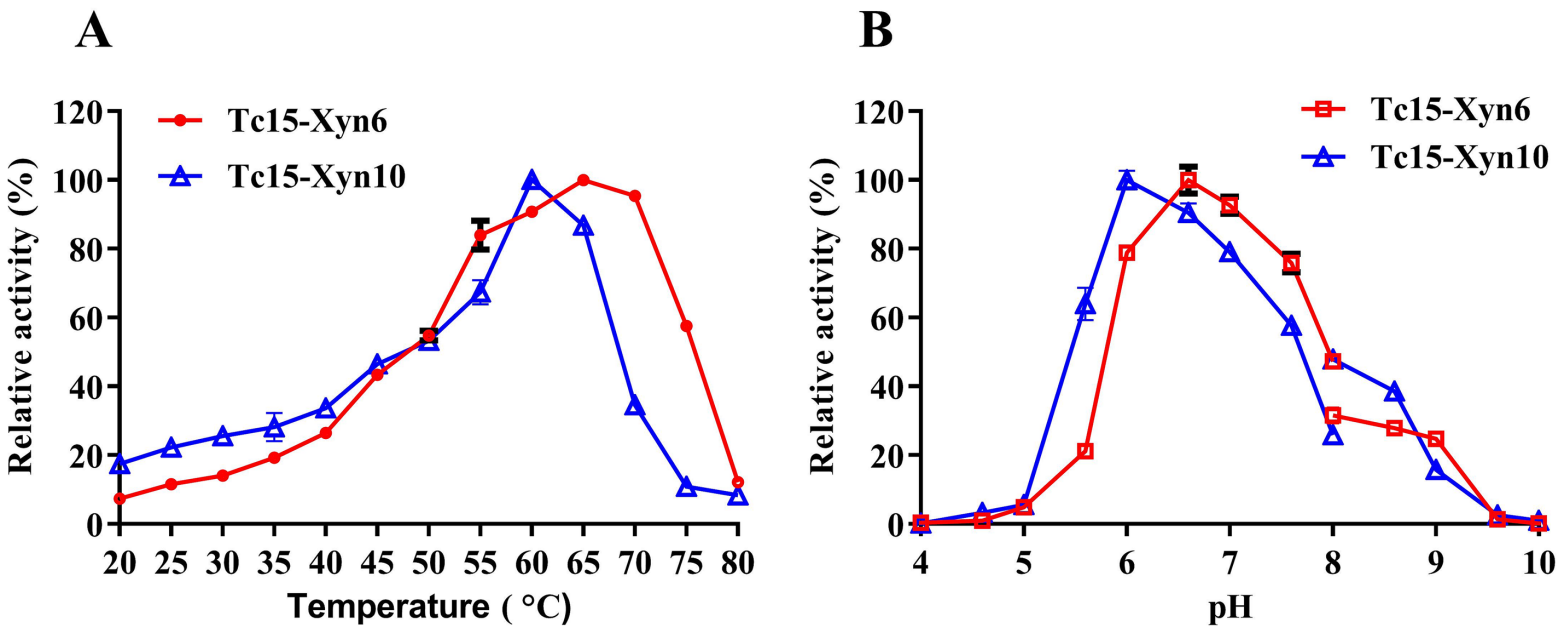
Optimal temperature **(A)** and optimal pH **(B)** of Tc15-Xyn6 and Tc15-Xyn10.

**Figure 5:**
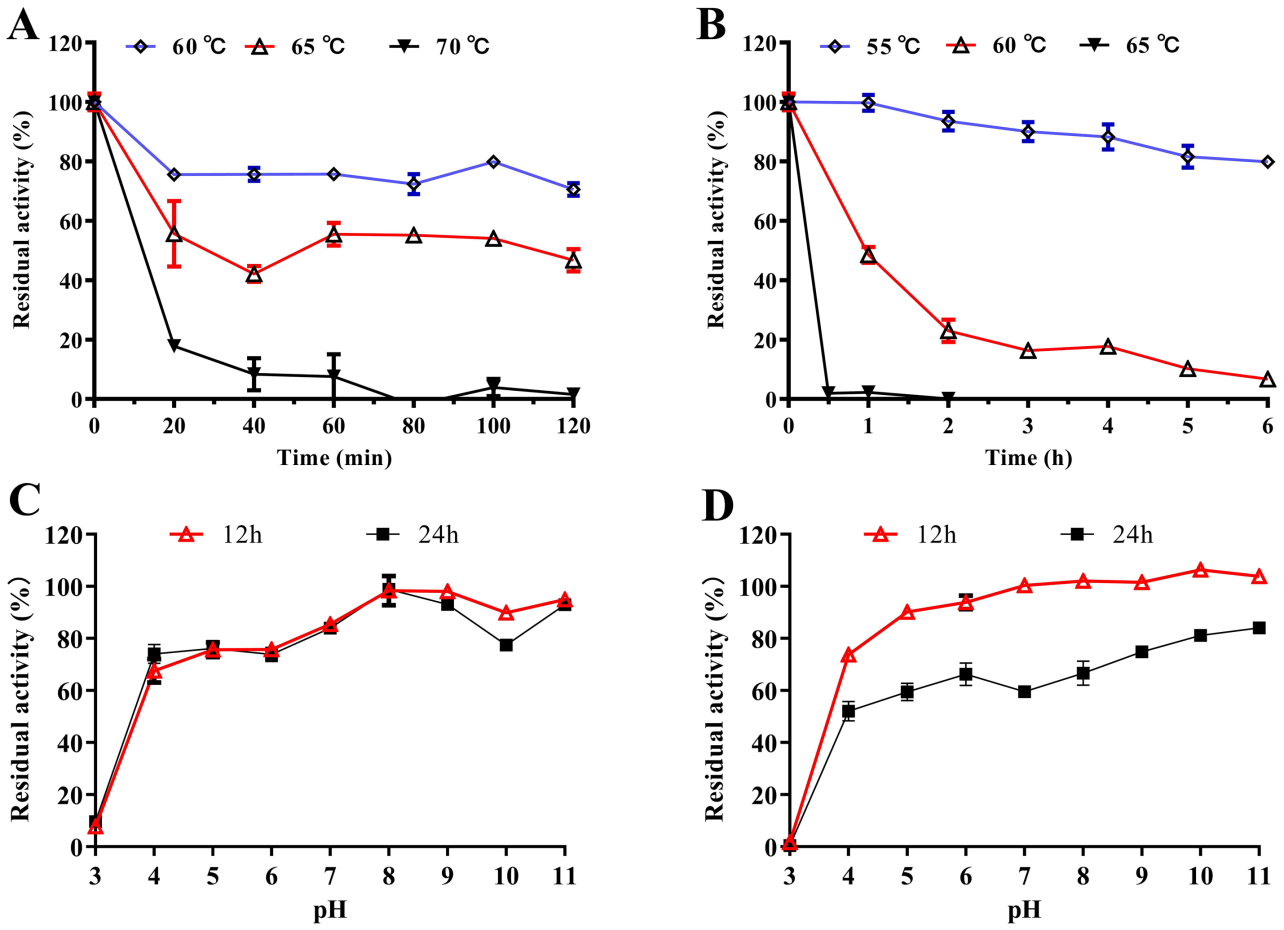
Stability of recombinant xylanases. **(A)** Thermal stability of Tc15-Xyn6; **(B)** thermal stability of Tc15-Xyn10; **(C)** pH stability of Tc15-Xyn6; **(D)** pH stability of Tc15-Xyn10.

Tc15-Xyn6 exhibited an optimal temperature of 65 °C and retained >54% relative activity between 55 and 75 °C. Tc15-Xyn10 showed an optimal temperature of 60 °C and maintained >60% relative activity between 55 and 65 °C (Figure 4A). Tc15-Xyn6 retained ~80% activity after 2 h at 60 °C; its half-life at 65 °C was ~2 h, and activity dropped to ~20% after 20 min at 70 °C. Tc15-Xyn10 retained ~80% activity after 6 h at 55 °C; its half-life at 60 °C was ~1 h, and activity decreased to ~2% after 30 min at 65 °C (Figures 5A,B).

#### pH optima and stability

The optimal pH was 6.6 for Tc15-Xyn6 (with >70% relative activity across pH 6.0–7.0) and 6.0 for Tc15-Xyn10 (with ~60% relative activity across pH 5.6–7.6) (Figure 4B). After incubation at 4 °C for 12 h and 24 h, Tc15-Xyn6 retained >60% of initial activity across pH 4.0–10.0. Tc15-Xyn10 retained >80% activity across pH 5.0–10.0 at 12 h and >40% activity across pH 4.0–10.0 at 24 h (Figures 5C,D).

### Effects of metal ions and chemical reagents on recombinant xylanase activity

**Figure 6:**
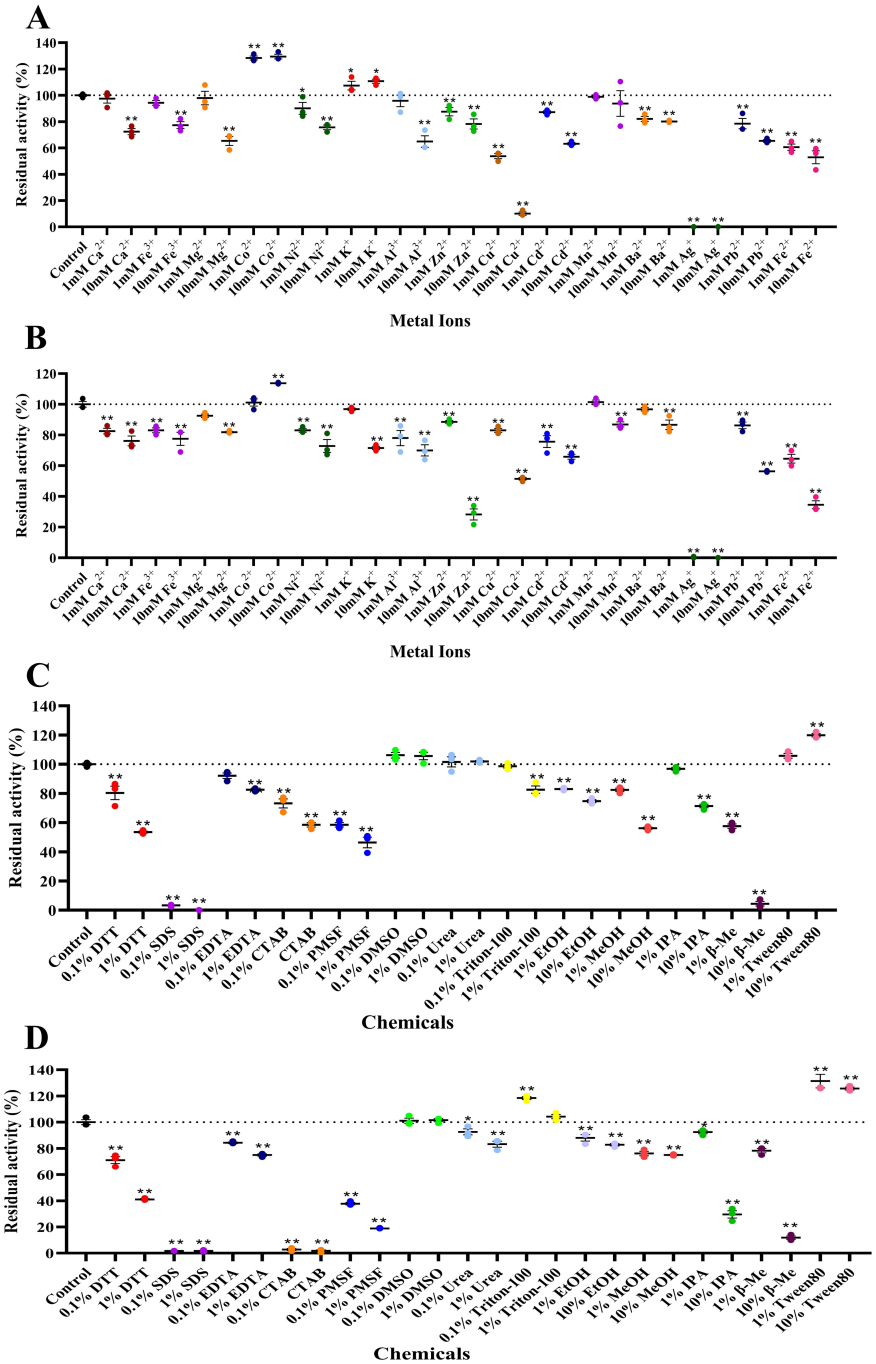
Effects of metal ions and chemical reagents on xylanase activity. **(A)** Tc15-Xyn6 with various metal ions; **(B)** Tc15-Xyn10 with various metal ions; **(C)** Tc15-Xyn6 with various reagents; **(D)** Tc15-Xyn10 with various reagents. Activity without additives was set to 100%. Values are mean ± SD. *p ≤ 0.05, **p ≤ 0.01 versus control.

At both 1 mM and 10 mM, Ag^+^ strongly inhibited both enzymes, whereas Co^2+^ showed an activating effect. For Tc15-Xyn6, activity increased in the presence of K^+^, but 10 mM Cu^2+^ reduced relative activity to ~10%; most other ions had weak effects. For Tc15-Xyn10, relative activity decreased to ~34.55% with 10 mM Fe^2+^ and ~28.26% with 10 mM Zn^2+^, while inhibition by most other ions was <50%. Among chemical reagents, SDS, β-mercaptoethanol, and PMSF significantly inhibited both enzymes. Tween 80 and DMSO showed activation, and Triton X-100 also activated Tc15-Xyn10. At 10% (v/v), isopropanol or DTT reduced Tc15-Xyn10 activity to <50%, and CTAB strongly inhibited activity. Notably, both enzymes retained >50% activity in 10% (v/v) methanol or ethanol, indicating a degree of alcohol tolerance (Figure 6).

### Substrate specificity and kinetic analysis

Substrate specificity was evaluated using multiple polysaccharides (Table 1). Tc15-Xyn6 showed a specific activity of 90.80 μmol/min/mg on beechwood xylan, and 46.84, 18.06, and 30.51 μmol/min/mg on corncob xylan, bagasse xylan, and CMC-Na, respectively. No activity was detected on Avicel or cellobiose. Tc15-Xyn10 exhibited 34.91 μmol/min/mg on beechwood xylan, and 14.68, 6.75, and 8.26 μmol/min/mg on corncob xylan, bagasse xylan, and Avicel, respectively; no activity was detected on CMC-Na or cellobiose. Thus, both enzymes preferentially hydrolyze xylan substrates from various sources, with minimal or no activity on cellulosic substrates and cellobiose.

**Table 1:** Specific activities (μmol/min/mg) of Tc15-Xyn6 and Tc15-Xyn10 on different substrates.

Substress	Specific activity (μmol/min/mg)	
	Tc15-Xyn6	Tc15-Xyn10
Beechwood xylan	90.80 ± 6.06^a^	34.91 ± 2.55^a^
Corncob xylan	46.84 ± 7.92^b^	14.68 ± 2.22^b^
CMC	30.51 ± 7.10^c^	0^d^
Bagasse xylan	18.06 ± 1.12^d^	6.75 ± 0.261^c^
Avicel cellulose	0^e^	8.26 ± 0.477^c^
Cellobiose	0^e^	0^d^

The same lowercase letters indicate no significant difference, while different lowercase letters indicate a significant difference (*p* < 0.05).

Kinetic analysis with beechwood xylan as substrate showed that Tc15-Xyn6 had *K*_m_ = 4.675 mg/mL, *V*_max_ = 125 μmol/min/mg, and *K*_cat_ = 93 s^−1^, whereas Tc15-Xyn10 had *K*_m_ = 9.36 mg/mL, *V*_max_ = 59.52 μmol/min/mg, and *K*_cat_ = 35.55 s^−1^ (Table 2). These results indicate higher substrate affinity and catalytic efficiency for Tc15-Xyn6 relative to Tc15-Xyn10.

**Table 2:** Kinetic parameters of Tc15-Xyn6 and Tc15-Xyn10 for beechwood xylan.

Parameters	Tc15-Xyn6	Tc15-Xyn10
Substrate	Beechwood xylan	Beechwood xylan
Optimal temperature	65 °C	60 °C
Optimal pH	6.6	6
Specific activity	90.80 ± 6.06	34.91 ± 2.55
*K*_m_	4.675 mg/ml	9.36 mg/ml
*V*_max_	125 μmol/min/mg	59.52 μmol/min/mg
Molecular mass	44.64KDa	35.84KDa
*K*_cat_	93 s^−1^	35.55 s^−1^

### TLC analysis of hydrolysis products

**Figure 7:**
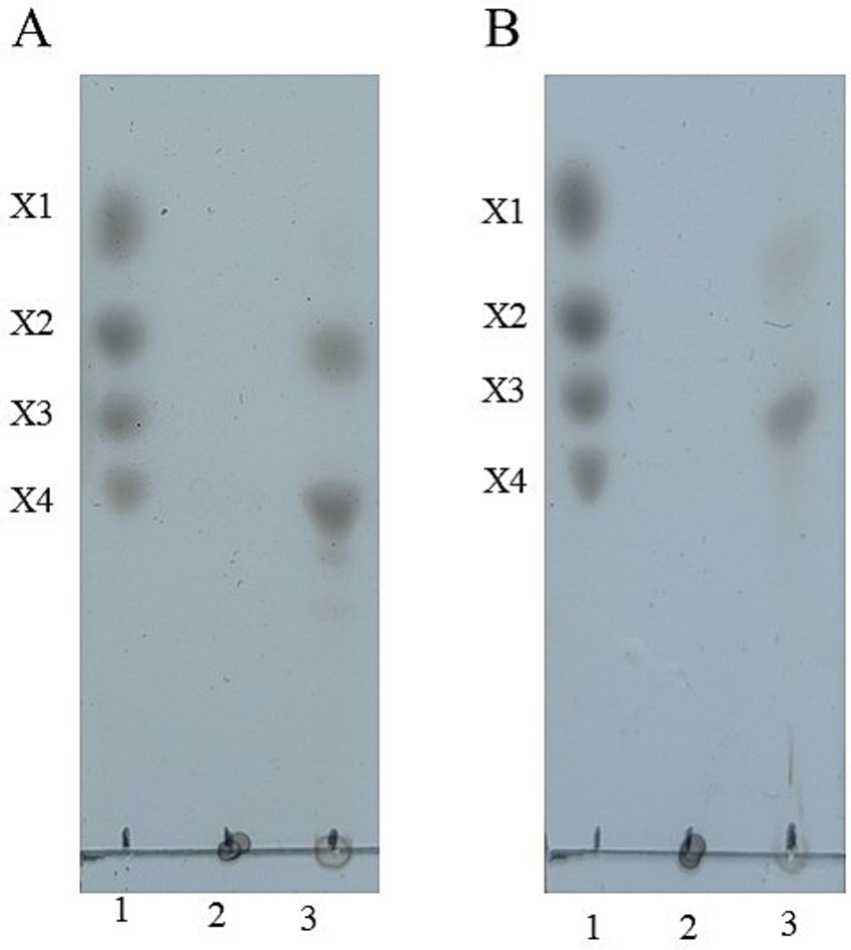
TLC analysis of hydrolysis products from beechwood xylan by Tc15-Xyn6 **(A)** and Tc15-Xyn10 **(B)**. Lane 1: XOS standards (X1 xylose, X2 xylobiose, X3 xylotriose, X4 xylotetraose). Lane 2: Inactivated enzyme + substrate. Lane 3: Active enzyme + substrate.

Using beechwood xylan as substrate, TLC revealed that Tc15-Xyn6 primarily produced xylobiose (X2) and xylotetraose (X4), whereas Tc15-Xyn10 predominantly generated xylotriose (X3) (Figure 7). No corresponding oligosaccharide bands were observed in inactivated enzyme controls or substrate blanks, confirming the enzymatic origin of the products.

### Enzymatic hydrolysis performance on high-temperature alkali-treated and high-temperature water-treated substrates

Both enzymes yielded low reducing sugar levels from poplar and pine residues. Among eight hot water–pretreated substrates, most produced <2 μmol/mL reducing sugars, with wheat bran exceeding 4 μmol/mL. By contrast, among eight hot alkali–pretreated substrates, wheat straw, rice straw, and corn stover each produced >2 μmol/mL (data not shown). Based on these observations, hot water–pretreated wheat bran and hot alkali–pretreated wheat straw, rice straw, and corn stover were selected for 48 h time-course analysis.

**Figure 8:**
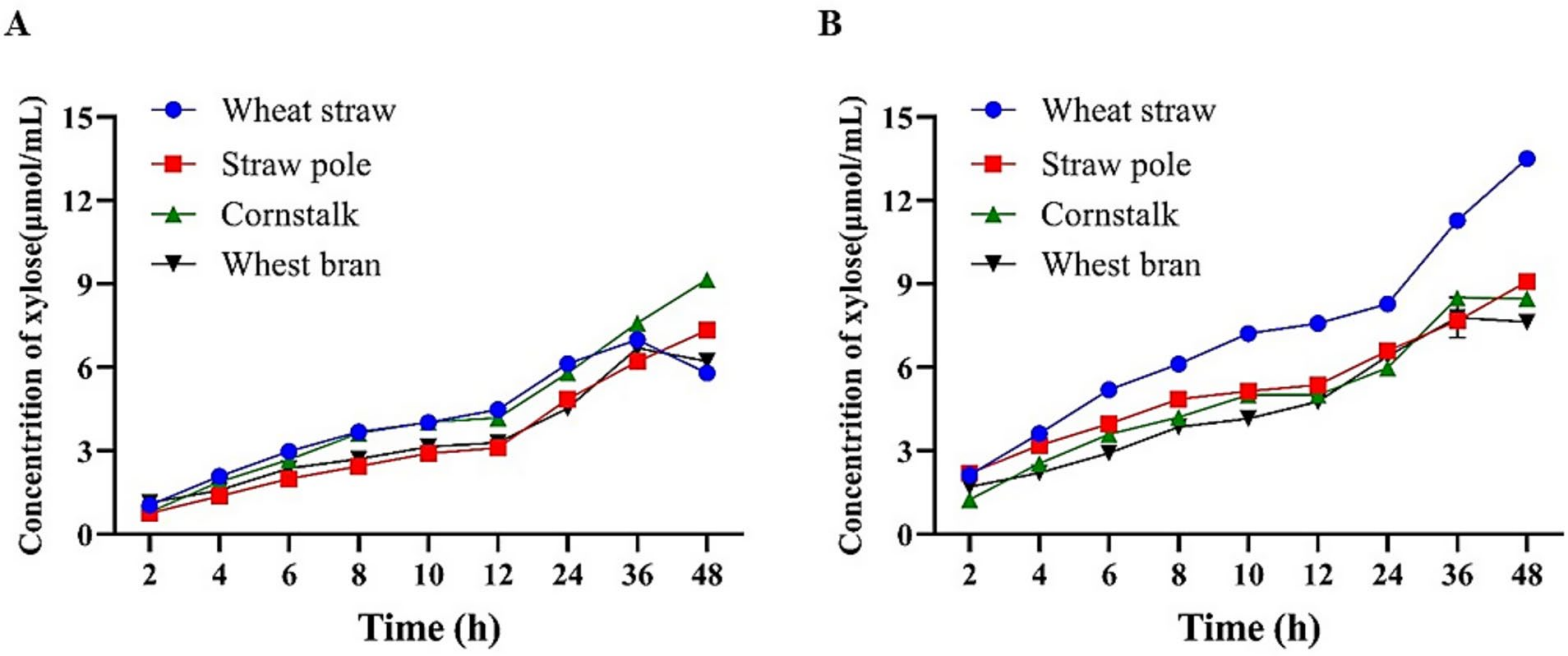
Reducing sugar production from different pretreated substrates over time. **(A)** Tc15-Xyn6; **(B)** Tc15-Xyn10.

For Tc15-Xyn6, reducing sugar production showed similar trends across the four substrates: prior to 36 h, wheat straw and corn stover yielded comparable, higher sugar levels than rice straw and wheat bran. At 36 h, wheat straw and wheat bran reached peak yields and then slightly declined, while rice straw and corn stover continued to increase (Figure 8A). For Tc15-Xyn10, prior to 36 h, sugar yields from rice straw, corn stover, and hot water–pretreated wheat bran were all lower than from wheat straw. By 48 h, yields from wheat bran and rice straw tended to stabilize, while those from corn stover and wheat straw continued to rise (Figure 8B). These results suggest that hot alkali–pretreated wheat straw and hot water–pretreated wheat bran are relatively suitable direct hydrolysis substrates for both enzymes.

**Figure 9:**
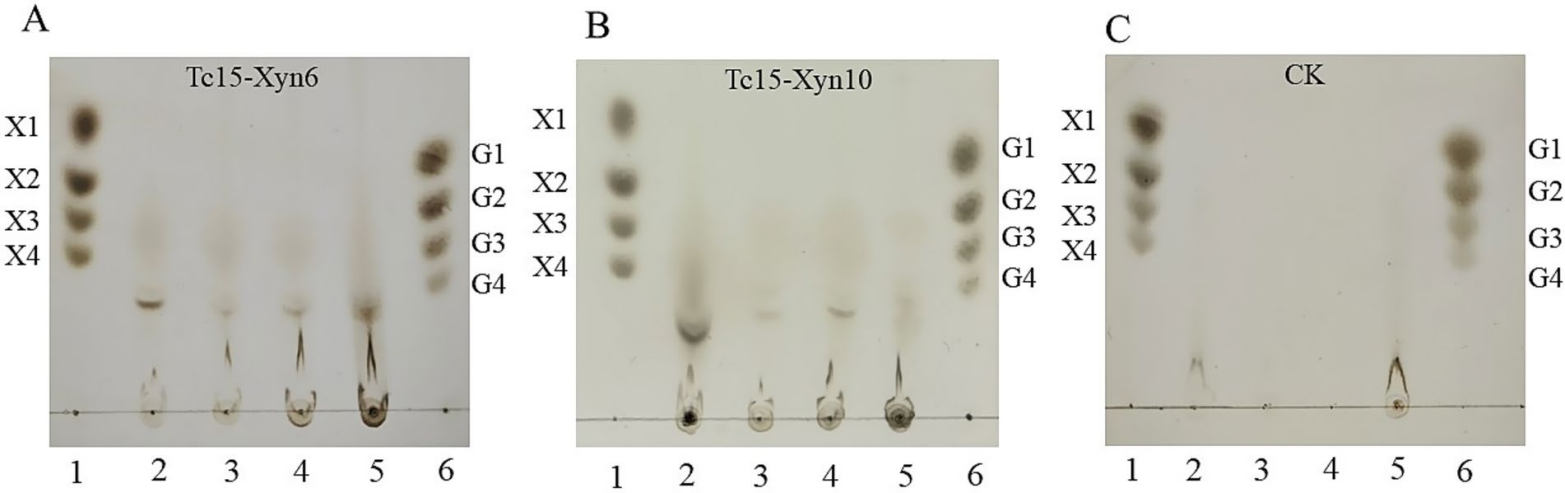
TLC of products after 48 h enzymatic hydrolysis of four representative substrates by Tc15-Xyn6 and Tc15-Xyn10. **(A)** Tc15-Xyn6; **(B)** Tc15-Xyn10; **(C)** Substrate blanks. Lane 1 **(A–C)**: XOS standards (X1–X4). Lane 6 **(A–C)**: Cellooligosaccharide standards (G1 glucose, G2 cellobiose, G3 cellotriose, G4 cellotetraose). For A and B, Lane 2, hot alkali–pretreated wheat straw; Lane 3, hot alkali–pretreated rice straw; Lane 4, hot alkali–pretreated corn stover; Lane 5, hot water–pretreated wheat bran. For C, Lanes 2–5, corresponding substrate blanks (sterile water + substrate).

Further TLC analysis of 48 h hydrolysates from the four representative substrates (hot alkali–pretreated wheat straw, rice straw, and corn stover; hot water–pretreated wheat bran) revealed xylooligosaccharide bands of higher degrees of polymerization for both enzymes, with no cellooligosaccharide (G2–G4) bands detected (Figure 9). This indicates no significant cellulolytic activity under the tested conditions.

### Analysis of the growth-promoting effects of prebiotic products

**Figure 10:**
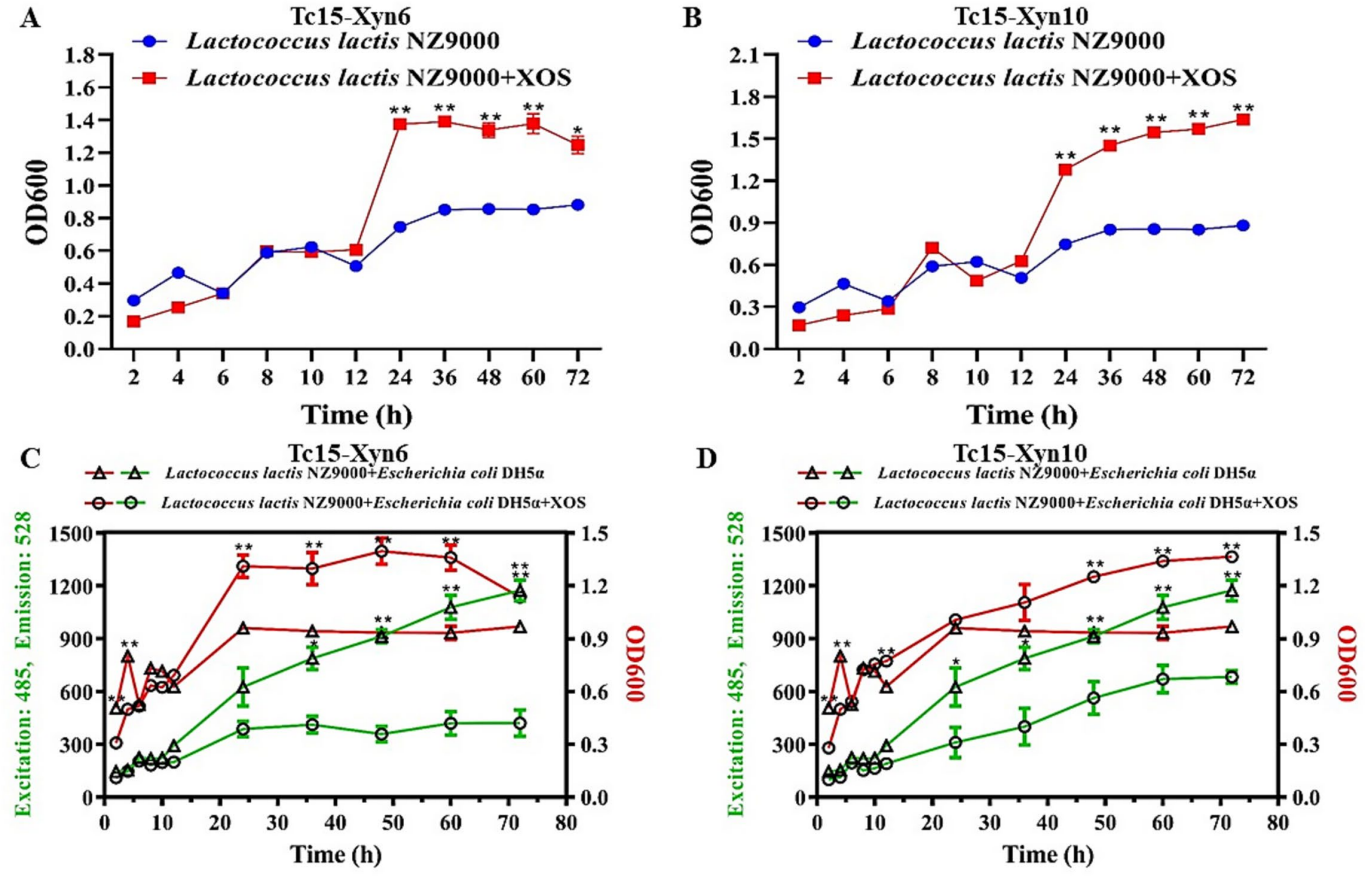
Growth-promoting effects of xylan hydrolysis products (prebiotics) from Tc15-Xyn6 and Tc15-Xyn10 on *Lactococcus lactis* and in co-culture. **(A)** Growth curve of *L. lactis* with Tc15-Xyn6 prebiotics. **(B)** Growth curve of *L. lactis* with Tc15-Xyn10 prebiotics. **(C)** OD600 and EGFP fluorescence in co-culture with Tc15-Xyn6 prebiotics. **(D)** OD600 and EGFP fluorescence in co-culture with Tc15-Xyn10 prebiotics. *P < 0.05 indicates a significant difference; **P < 0.01 indicates an extremely significant difference.

Xylan hydrolysates generated by Tc15-Xyn6 and Tc15-Xyn10 promoted the growth of *Lactococcus lactis*. In the control without prebiotics, *L. lactis* displayed a typical growth pattern: a lag phase during the first 12 h, entry into exponential growth between 12 and 36 h, and stabilization thereafter (Figures 10A,B). In cultures supplemented with Tc15-Xyn6-derived prebiotics, growth accelerated between 12 and 24 h, and biomass stabilized at higher OD600 values from 24 to 48 h relative to the control. With Tc15-Xyn10-derived prebiotics, *L. lactis* exhibited a sustained increase over 12–72 h, with a pronounced acceleration between 12 and 36 h, followed by a moderate rise from 36 to 72 h (Figures 10A,B).

Co-culture assays with EGFP-labeled *E. coli* and *L. lactis NZ9000* were used to simulate gut-like competitive dynamics. In the control, EGFP fluorescence increased continuously, indicating progressive *E. coli* expansion and concomitant suppression of *lactic acid bacteria*. In contrast, Tc15-Xyn6 prebiotics produced a marked increase in total biomass (OD600) and a substantially lower fluorescence signal than the control, consistent with a higher *L. lactis* abundance and reduced *E. coli* proliferation. Tc15-Xyn10 prebiotics similarly elevated OD600 while lowering EGFP fluorescence relative to the control, indicating increased *lactic acid bacteria* counts and reduced *E. coli* abundance (Figures 10C,D). Collectively, these results demonstrate that Tc15-Xyn6- and Tc15-Xyn10-derived xylan hydrolysates act as prebiotics that selectively promote *lactic acid bacteria* growth and favorably modulate competitive community dynamics.

## Discussion

In this study, two GH10 xylanases, Tc15-Xyn6 and Tc15-Xyn10 were identified from the metagenome of microorganisms inhabiting Tengchong Qiaoquan hot spring sediments. Following cloning, heterologous expression, and purification, both enzymes were characterized as thermophilic and thermostable, with Tc15-Xyn6 displaying an optimal temperature of 65 °C and a half-life of ~2 h at 65 °C, and Tc15-Xyn10 exhibiting an optimal temperature of 60 °C and a half-life of ~1 h at 60 °C while retaining ~80% activity after 6 h at 55 °C. These profiles support operation at moderate to elevated temperatures, a key requirement for lignocellulose bioprocessing and prebiotic XOS production.

Phylogenetically, Tc15-Xyn6 clustered with Blastocatellia and Tc15-Xyn10 with Meiothermus. Homology modeling using SWISS-MODEL yielded high-identity templates (Tc15-Xyn6: 77.10%; Tc15-Xyn10: 83.33%) with strong GMQE scores (0.94 and 0.97, respectively). Both proteins adopt the canonical GH10 (*α*/*β*)8 TIM-barrel architecture, in which the active site is positioned at the C-terminus of the β-strands. The prevalence of β-sheet content and potential salt-bridge formation are widely associated with enhanced fold stability and thermostability (Huang et al., 2023), consistent with the measured thermal resilience of both enzymes.

### Industrial significance of temperature, pH, and process tolerance

From a deployment perspective, temperature and pH optima critically determine enzyme operability and cost effectiveness on complex, partially pretreated substrates and under non-sterile conditions. While many reported xylanases exhibit optima between 30 and 60 °C and pH 4.0–9.0 (Dhiman and Mukherjee, 2018), representative neutral-preferring, moderately thermophilic enzymes such as AWS-2X, PW-xyl9, and PW-xyl37 operate at 55–60 °C (Wang et al., 2017, 2021). Tc15-Xyn6 and Tc15-Xyn10 show superior stability in the 55–65 °C range: Tc15-Xyn6 has a half-life of ~2 h at 65 °C, and Tc15-Xyn10 has a half-life of ~1 h at 60 °C, with substantial activity retention at slightly lower temperatures. These properties are advantageous for XOS production: operation at 55–60 °C improves xylan solubility and mass transfer, reduces viscosity, and suppresses microbial contamination, enabling stable continuous or semi-continuous processes without stringent sterilization. Tc15-Xyn6’s tolerance to short exposures at even higher temperatures provides flexibility for intensified hydrolysis with high substrate loading and short residence times.

Both enzymes exhibit neutral pH preference and broad pH tolerance. The optimal pH values were 6.6 (Tc15-Xyn6) and 6.0 (Tc15-Xyn10), with Tc15-Xyn6 retaining >60% activity across pH 4.0–10.0 and Tc15-Xyn10 retaining >50% activity across pH 5.0–10.0. This broad “stability window,” reported for several GH10 xylanases (e.g., Xyl10B, Xyn30Y5-SLH, Af-XYLA) (Bernardi et al., 2021; Lai et al., 2021; Mon et al., 2022), is valuable for food-grade XOS production. Operating near neutral pH (5–7) minimizes acid/base consumption and neutralization costs, mitigates sugar degradation (e.g., furfural formation), and facilitates integration with mild upstream pretreatments (hot water or dilute alkali).

### Substrate specificity, kinetics, and product profiles

Both enzymes preferentially hydrolyzed xylan from multiple sources and showed negligible activity on Avicel (Tc15-Xyn6) or CMC-Na (Tc15-Xyn10), with no activity detected on cellobiose. On beechwood xylan, Tc15-Xyn6 exhibited higher catalytic efficiency and affinity (*K*_m_ 4.675 mg/mL; *V*_max_ 125 μmol/min/mg; *K*_cat_ 93 s^−1^) than Tc15-Xyn10 (*K*_m_ 9.36 mg/mL; *V*_max_ 59.52 μmol/min/mg; *K*_cat_ 35.55 s^−1^). The study revealed that the *K*_m_ and *V*_max_ values of CrXyn toward beechwood xylan were 5.98 g/L and 179.9 μmol/min/mg, respectively, while those of XynB for the same substrate were 5.82 mg/mL and 380 μmol/min/mg (Zhu et al., 2022; Guo et al., 2013). The *K*_m_ value of Tc15-Xyn6 was essentially comparable to those of the aforementioned enzymes, although XynB exhibited a higher *V*_max_; overall, Tc15-Xyn6 displayed substrate affinity similar to that of other reported xylanases. For BhS7Xyl, its *K*_m_ and *V*_max_ toward beechwood xylan were determined to be 32.1 mg/mL and 41.9 μmol/min/mg. Tc15-Xyn10 showed superior overall catalytic properties compared to BhS7Xyl but was inferior to Tc15-Xyn6. Xiao characterized the polysaccharide CVP-2 derived from *Christia vespertilionis* and demonstrated that its branched chain structures (e.g., *α*-l-arabinofuranosyl branches) can enhance its binding capacity to biological substrates, while the stable backbone structure (→5-α-l-arabinofuranosyl) ensures structural stability during the catalytic process (Xiao et al., 2025). It is speculated that the *K*_m_ values and *V*_max_ of Tc15-Xyn6 and Tc15-Xyn10 toward xylan may be associated with similar branched chain structural characteristics and structural stability.

TLC profiles revealed enzyme-specific XOS signatures: Tc15-Xyn6 predominantly yielded xylobiose (X2) and xylotetraose (X4), whereas Tc15-Xyn10 produced xylotriose (X3). Such product selectivity is important for tailoring XOS composition to target microbial beneficiaries and functional outcomes in feeds or functional foods.

### Performance on pretreated biomass and prebiotic evaluation

Hydrolysis of pretreated substrates showed that hot alkali–pretreated wheat straw and hot water–pretreated wheat bran produced the highest sugar yields across time courses, supporting their use as practical feedstocks. The absence of detectable cellooligosaccharides by TLC suggests minimal cellulase activity under test conditions, which is desirable for XOS-centric processes that aim to limit glucose release and downstream interference.

Crucially, the resulting XOS-rich hydrolysates significantly promoted *L. lactis* growth and shifted co-culture dynamics toward l*actic acid bacteria* dominance by suppressing *E. coli* expansion. These results corroborate the prebiotic potential of Tc15-derived XOS and highlight their suitability for functional feed applications that aim to support gut health and reduce opportunistic pathogens.

### Process compatibility informed by metal ions and chemical factors

The influence of metal ions and chemical additives on enzyme activity provides essential guidance for process design and upstream media purification. Both Tc15-Xyn6 and Tc15-Xyn10 were strongly inhibited by Ag^+^, consistent with the broad-spectrum antimicrobial and protein-denaturing properties of silver ions (Qadeer et al., 2024). It is plausible that Ag^+^ interacts with acid/base catalytic residues in the active site or with structural elements critical for proper folding, inducing conformational perturbations that hinder substrate binding. Although Ag^+^ contamination is uncommon in food-grade or conventional biomanufacturing systems, care should be taken to avoid contact with silver-containing antimicrobial materials or disinfectants; if necessary, trace heavy metals should be removed via water pretreatment, chelation, or adsorption.

SDS also strongly inhibited both enzymes, consistent with its function as an anionic denaturant that disrupts native protein conformations (Parker and Song, 1992; Srivastava and Alam, 2020; Krishnamani et al., 2012; Lefebvre-Cases et al., 2001; Minai-Tehrani et al., 2012). While SDS is unlikely to be present in food-grade processes, these results underscore the need to avoid contamination by strong surfactants and detergents. Notably, both xylanases retained more than 50% of their activity in 10% (v/v) methanol or ethanol, demonstrating tolerance to polar small-molecule alcohols (Benatti and Polizeli, 2023). This compatibility facilitates integration with solvent-based pretreatments (e.g., organosolv delignification or hemicellulose separation) and alcohol-containing steps (e.g., ethanol washing for lignin removal, or residual ethanol at the end of fermentation), and supports cross-application in alcohol-involving industries such as papermaking and biofuels.

### Molecular docking insights into product profiles and stability

**Figure 11:**
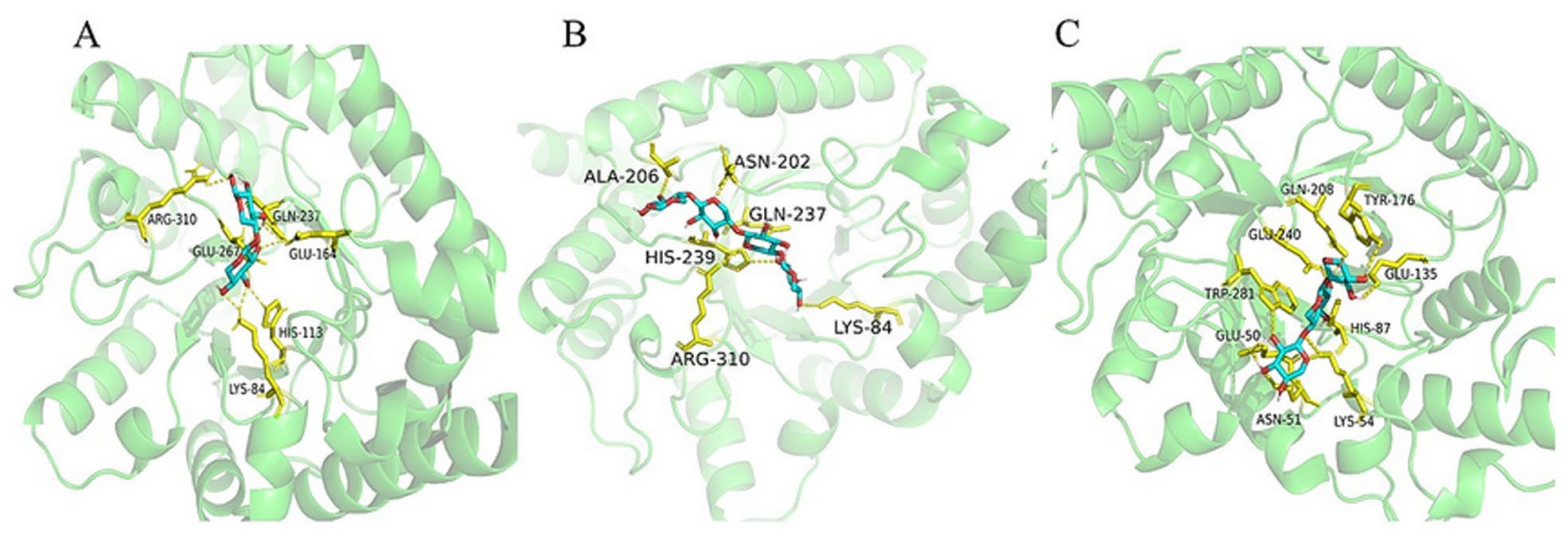
Molecular docking analysis. **(A)** Tc15-Xyn6 with xylobiose (X2). **(B)** Tc15-Xyn6 with xylotetraose (X4). **(C)** Tc15-Xyn10 with xylotriose (X3).

Enzyme–ligand docking was performed using AutoDock Tools 1.5.6 (ADT) and visualized in PyMOL (Ellilä et al., 2019). The results provide structural rationales for the observed product selectivity and stability profiles. For Tc15-Xyn6, minimum binding energies were −6.7 kcal/mol for xylobiose (X2) and −8.1 kcal/mol for xylotetraose (X4). Hydrogen-bonding interactions for X2 involved Lys84, His113, Glu164, Gln237, Glu267, and Arg310; for X4 they involved Lys84, Asn202, Ala206, His239, and Arg310. For Tc15-Xyn10, the minimum binding energy for xylotriose (X3) was −8.7 kcal/mol, with hydrogen bonds primarily mediated by Glu50, Asn51, Lys54, His87, Glu135, Tyr176, Gln208, Glu240, and Trp281 (Figure 11). Consistent with Hassan et al. (2023), biomolecular structural stability is closely linked to the strength and organization of intramolecular interactions. Covalent disulfide bonds can rigidify protein conformations, while strengthened hydrophobic packing limits exposure of nonpolar cores and mitigates heat-induced unfolding. In the present study, *in silico* analyses predict that extensive hydrogen-bond networks—together with complementary hydrophobic contacts and potential salt bridges—attenuate thermal denaturation, providing a plausible molecular basis for the observed thermostability of the xylanases. Dense hydrogen-bond networks are associated with increased protein rigidity and enhanced conformational stability at elevated temperatures (Günther et al., 2023; Su et al., 2021). We hypothesize that the number of hydrogen bonds may affect the structural stability of the enzyme, resulting in differences in the thermotolerance of xylanases. Temperature tolerance experiments demonstrated that Tc15-Xyn10 maintained more than 80% activity after 6 h at 55 °C, whereas Tc15-Xyn6 decreased to approximately 36% after 6 h at 55 °C (Supplementary Figure S1). Molecular docking suggests the number of hydrogen bonds formed in Tc15-Xyn10 is 4/3 times that in Tc15-Xyn6. Tc15-Xyn10 forms a greater number of hydrogen bonds relative to Tc15-Xyn6, consistent with the observed long-term stability of Tc15-Xyn10 at 55 °C. Conversely, Tc15-Xyn6 exhibits superior short-term thermostability at higher temperatures, aligning with its kinetic performance and product profile. Both temperature tolerance experiments and molecular docking have indirectly verified that the number of hydrogen bonds influences the thermotolerance of xylanases; however, to fully clarify the role of hydrogen bonds, further validation through molecular dynamics simulations and mutagenesis experiments is required.

### Feasibility and application scenarios for XOS production from natural straw substrates

The dense architecture of lignocellulose and tight cross-linking between lignin and hemicellulose limit xylanase accessibility and hydrolysis efficiency. Appropriate pretreatment is therefore essential (Ramesh et al., 2018; Alawad and Ibrahim, 2022). In this study, wheat bran, wheat straw, rice straw, and corn stover were subjected to high-temperature (HT) and high-temperature alkaline (HTA) pretreatments. Results were consistent with established consensus: alkaline pretreatment promotes delignification, cleaves ester bonds and acetyl groups, and increases porosity through cell wall swelling, thereby improving hemicellulose accessibility and xylanase performance (Wu X. et al., 2022; Wu Z. et al., 2022). Compared with high-temperature water-only pretreatment, HTA substrates exhibited looser structures that enabled Tc15-Xyn6 and Tc15-Xyn10 to release more reducing sugars. Both enzymes also showed excellent hydrolysis on high-temperature water-pretreated wheat bran, suggesting that for raw materials rich in soluble or weakly cross-linked xylan (e.g., wheat bran), simple HT pretreatment can provide sufficient accessibility for neutral xylanases.

Relative to reported enzymatic XOS production from straw (e.g., XynB for garlic straw, Xyln-SH1 for wheat straw, MxynB-8 for poplar sawdust, as detailed in Table 3) Tc15-Xyn6 and Tc15-Xyn10 offer three practical advantages: (1) Neutral pH optima with moderate-to-high temperature tolerance, facilitating integration with mild pretreatments and reducing neutralization and cooling costs. (2) Alcohol tolerance, enhancing compatibility with alcohol-containing pretreatments and solvent recycling. (3) Product spectra dominated by X2–X4, aligning with desirable prebiotic XOS profiles.

**Table 3:** Illustrative comparison of straw-to-XOS production by xylanases.

Enzyme	Sources	GH family	Optimal condition	Zymolyte	Pre-processing method	XOS Production	Reference
Tc15-Xyn6	Hot Spring Metagenome	GH10	pH6.6, 65 °C	Wheat straw^1^ Straw pole^2^	1, 2, 3 alkali method	XOS(>X4)	This study
Tc15-Xyn10	Hot Spring Metagenome	GH10	pH6.0, 60 °C	Cornstalk^3^ Wheat bran^4^	4 hydrothermal method	XOS(>X4)	This study
Xyn B	*B. mojavensis*	GH11	pH4.0, 50 °C	Garlic straw	The alkali extraction	X2, X3	Günther et al. (2023)
Xyln-SH1	Holstein cattle rumen metagenomic library	GH10	pH6.5, 40 °C	Wheat straw	Alkali method	XOS	Su et al., 2021
MxynB-8	*Aspergillus niger* NL-1	GH11	pH6.0, 50 °C	Poplar sawdust	Alkali method	X2, X3	Kallel et al. (2015)

It is important to emphasize that straw type, pretreatment conditions, and solids loading strongly influence hydrolysis. Future work should systematically evaluate yield, product selectivity, and energy consumption at higher solids (>10–15% w/w) and under continuous or semi-continuous operation to ensure comparability with published processes and identify optimal operating windows.

### Synergistic benefits for straw valorization and feed applications

Xylanase treatment mitigates anti-nutritional effects of dietary xylan, disrupts cell wall structures, and frees encapsulated starch, proteins, and micronutrients, thereby enhancing digestibility in monogastric animals (Adebowale, 1985; Zhang et al., 2012; Bimrew, 2014). Wheat bran, a major feed byproduct, is rich in NSPs—especially xylan—that increase digesta viscosity, hinder enzyme–substrate contact, and impair nutrient absorption (Swiatkiewicz et al., 2015). Tc15-Xyn6 and Tc15-Xyn10 showed strong hydrolysis of HT-treated wheat bran, highlighting their potential for targeted enzymatic modification. Two application strategies emerge: (1) In-plant enzymatic pretreatment to partially hydrolyze bran, yielding “functionalized wheat bran” or blended liquid/wet feed ingredients with other byproducts. (2) Direct inclusion in diets, leveraging gastrointestinal pH (5–7) for optimal activity. Given transient high temperatures during pelleting, the thermostability of Tc15-Xyn6 and Tc15-Xyn10 at 60–65 °C is advantageous; further tolerance can be pursued via encapsulation or carrier immobilization. Improved digestibility and nutrient release, coupled with reduced excretion of undigested material, are expected to lower environmental burdens in livestock and poultry production (Lian et al., 2024), promoting high-value utilization and a circular economy for straw byproducts.

### Prebiotic XOS: biological effects and prospects for functional products

Tc15-Xyn6 primarily produced X2 and X4, while Tc15-Xyn10 predominantly yielded X3; products were concentrated in the X2–X4 range. XOS within this DP range exhibit typical prebiotic traits—low effective dosage, high selectivity—and are efficiently utilized by Lactobacillus and Bifidobacterium (Maria et al., 2014). The XOS generated here promoted *L. lactis* growth and suppressed *E. coli*, consistent with reports that dietary XOS produced *in situ* by xylanases favor beneficial bacteria (e.g., *Streptococcus lactis*, *Lactobacillus bulgaricus*) and inhibit opportunistic pathogens (e.g., *Klebsiella pneumoniae*) (Ding et al., 2018; Kim et al., 2011; Yadav and Jha, 2019; Ramatsui et al., 2024). In poultry, in ovo injection of X2 and X3 improves growth and intestinal morphology (Palaniappan et al., 2021). In mice, XOS alleviated diarrhea via gut microbiota modulation (Singh et al., 2015). In human nutrition, XOS are incorporated into functional foods (e.g., dairy, baked goods) with reported immunomodulatory, preservative, and antioxidant effects (Prete et al., 2021; Kumar et al., 2025; Das et al., 2024). Collectively, these findings support a conversion pathway from raw biomass to enzymatic hydrolysate to XOS to functional products, with Tc15-Xyn6 and Tc15-Xyn10 as enabling biocatalysts.

## Conclusion

In summary, this study successfully identified and characterized two novel GH10 xylanases, Tc15-Xyn6 and Tc15-Xyn10, from the Qiaoquan hot spring in the Tengchong Rehai Geothermal Area using enrichment culture and metagenomic mining. After cloning and heterologous expression in *E. coli*, both enzymes were purified and comprehensively profiled. Tc15-Xyn6 displayed optimal activity at 65 °C and pH 6.6 with a half-life of ~2 h at 65 °C; Tc15-Xyn10 was optimal at 60 °C and pH 6.0 with a half-life of ~1 h at 60 °C and retained ~80% activity after 6 h at 55 °C. Both enzymes were inhibited by Ag^+^ and SDS yet tolerated 10% (v/v) methanol or ethanol. They efficiently hydrolyzed xylan from high-temperature alkali–treated straw (wheat, rice, and corn stover) and hot water–treated wheat bran, producing XOS dominated by X2–X4 that promoted *L. lactis* growth and favorably modulated co-culture dynamics against *E. coli*. Collectively, these attributes identify Tc15-Xyn6 and Tc15-Xyn10 as promising biocatalysts for the green conversion of lignocellulose to functional XOS, with potential applications as feed additives and in prebiotic production; nevertheless, further validation under industrial conditions (e.g., pilot-scale hydrolysis and cost-effectiveness analysis) is required.

## Data Availability

The nucleotide sequences of the thermophilic xylanase genes Tc15-Xyn6 and Tc15-Xyn10 from the Tengchong Qiaoquan hot spring have been deposited in the NCBI GenBank database under accession numbers PX522158 and PX522161, respectively.

## Footnotes

1
https://img.jgi.doe.gov/cgi-bin/mer/main.cgi

2
http://blast.ncbi.nlm.nih.gov/Blast.cgi

3
http://www.cbs.dtu.dk/services/signalp/
