# Supplementary material for: Characterization of thermophilic xylanases from Tengchong Qiaoquan hot spring for lignocellulose bioprocessing and prebiotic production
Source: Front Microbiol. 2026 Jan 23;16:1731615. doi: 10.3389/fmicb.2025.1731615 (PMC12880817; doi:10.3389/fmicb.2025.1731615)
Supplement: Supplementary file 1 [file Supplementary_file_1.docx]

Supplementary Material

## 1 Supplementary Figure


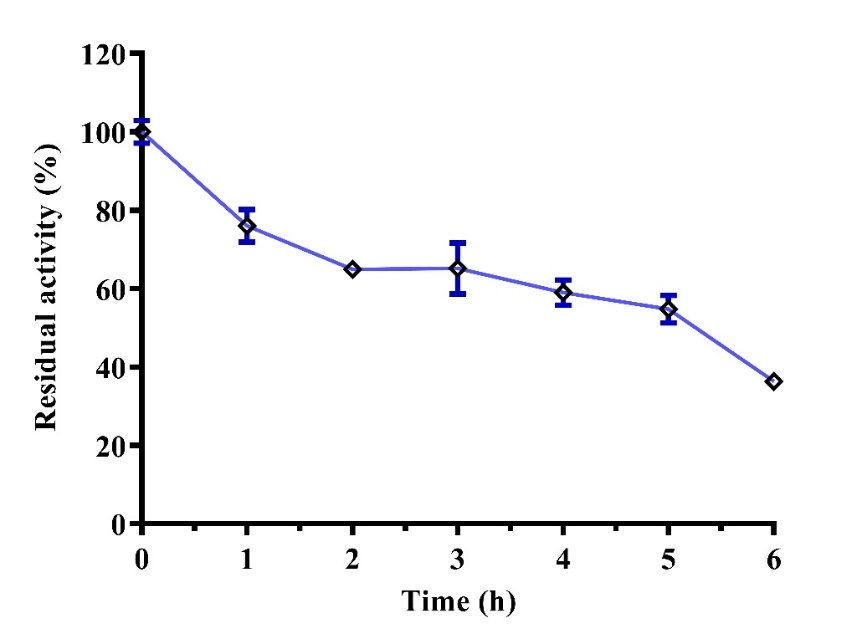


**Supplementary Figure 1.** Thermal stability profile of Tc15-Xyn6 at 55 °C.

## 2 Supplementary Table

**Supplementary Table 1.** Molecular docking parameters

| xylooligosaccharides | Molecular docking parameters |
| --- | --- |
| TC15-Xyn6-X2 | center_x = 2.312  center_y = 0.622  center_z = -2.721  size_x = 36.0  size_y = 36.0  size_z = 47.25 |
| TC15-Xyn6-X4 | center_x = 2.929  center_y = 2.969  center_z = -1.751  size_x = 34.5  size_y = 36.0  size_z = 47.25 |
| TC15-Xyn10-X3 | center_x = 3.206  center_y = -0.142  center_z = 2.583  size_x = 33.75  size_y = 34.5  size_z = 38.25 |

DNA Sequences

>*Tc15-Xyn6*

CAAGACTGGCGCGCCGCCGCCGAGGCGCGCATCGAGAGGATCCGCAAAGGCGAGCTTGTCGTGCAGGTGGAAGACCGCGACGGCAAGCCGCTGCCGGGCGTGCGGGTGGAGGTCCGCATGCTGCGCCATGCCTTCGGCTGGGGCACGGCGGTGTCGGCGAGGCAGCTGCTGGCCGAGGGCGCCGATGCCGAGCGCTACCGCCGCGCGGTTCTCGACAACTTCAACATGGTGGTGCTCGAAAACGACCTCAAGTGGCCGCAGTGGGAGCGCGACCGCCAGCCGGCGCTGGCCGCGCTGCGCTGGCTCCATGACAACGGGATTTCCCGCGTGCGCGGCCACACGCTCGTCTGGCCCGGCTGGCGCTGGCTGCCGCCGGACCTGAAGGAGCTGGCGGCCGATCCGGCGGCGCTGCGGAAGCGCGTGCTCGACCACATCCGCGACGAGGTCTCCGCCACGAGCGGCCTGCTCGAGGACTGGGACGTCGTCAACGAGCCCTACACCAACCACGACCTGCTGGACATTCTCGGCCGCGAGGAGATGGTTGCGTGGTACCAGGCGGCGAGGGAGTTCGACCCGAAGCCGGTCCTGTATCTGAACGACTTCAACATCATCGAAGCGGGCGGCCGGGACGAAAAGCACCGCCAGCATTTCTACGAGACCATCCGCTTCCTGCTCGAGCGCGGCGCGCCGCTGGGCGGCATCGGCATCCAGGGGCATTTCCGGGAGCCCACGCCGCCGGAGAAAATTCTCGAAATTCTCGATCTGTTCTCGGAATTCAACCTGCCGATCCGCATTACCGAATTCGATTTCGAGACGAAAGACGAACAGCTGCAGGCGGAATTCACCCGCGATTTTCTCACCCTCTGCTTCAGCCACCCGCGCGTGGACGCCTTCCTGATGTGGGGCTTCTGGGAAGGCCGGCACTGGCGGCCCGACGGTGCGATGCTGCGGAAGGACTGGTCGGAGAAGCCCGTTTACCGCGTCTGGCGGGAGCTCGTGCACGGCCGCTGGCGCACCGAAGCCGAGGGCGTCACCGGAGACGACGGCAGTTTCCGCCTGCGCGCCTTCCGTGGCGAGTACGAAATCCGCGCCGGCGGCCGCACGCTGCGCGCCGCCTGTCCGTCGGTAGTTCGTGTGCGGCTCGAG

>*Tc15-Xyn10*

ATGGCCCTGGCCCAGTCTGGCCCGCCCCTGCGCGCCCTGGCCGAAAAGCGCAATTTTCAGATTGGGGCTGCGGTGGAGCCCAGCCTGCTCCTGCAAGAGCCCGAGTACGCTCGGATCTTAGCCCAGGAGTTCAACCTGGTGGTGGCCGAAAACGTGATGAAGTGGGGGGCCTTGCAGACCTCCCGCGGCCAGTACAACTTTGCCCTGGCCGACCTGCTGATGGATTTTGCCCAGAAAAACCGCATGGCCGTGCGGGGGCATACCCTGGTCTGGCACCAACAGCTTCCCCGCTGGATGTACGGGGGTTTTAGCCCCGCCGAGATGGAGGCCATCCTGCGGGAGCATATTCAGACCGTGGTGGGGCGCTACCGGGGCCGGATCGCCTACTGGGATGTGGTCAACGAGGCGGTTGGGGACGACGCCAAGCTGCGCCCAACCCCTTTTGAGGTCTTGCCCGACTACCTGGAGAAGGCCTTCCGCCTGGCCCGTGAGGCCGACCCCCAGGCCAAGCTTTTCTACAACGACTACGGGGCCGAGGGGCTGGGCCCCAAGTCGGACGCCATCTACGCCCTGCTCAAAGGGCTGAAGGAAAGGGGCGTGCCCCTGGATGGGGTGGGCTTTCAGGTGCACGTGGACCTGGGCTTCTCGCCTGGGGCTGTGCGCATGGCGGAGAACCTCGAGCGCTTCGCCAGGCTGGGCCTGGAAATTCACATCACCGAGATGGACGTGCGGCTTGGTGGGCCTGGCAGCCGGGCCGAACGCTTGGAGAAGCAGGCCCAGGTGTACCGCGAGGTGATGCGGGTCTGCCTGGGCCAGCCCCGTTGCAAAGCCTTCACCCTTTGGGGCTTCACCGACGCCCACTCCTGGCGGTCGGCCAGCGAGCCCCTCATCTTCGACGCCGACTACAAACCTAAGCCTGCCTACCTTGCCCTACAGCAAGCCTTGCAACGACCCTGA

Proein Sequences

*>* Tc15-Xyn6

QDWRAAAEARIERIRKGELVVQVEDRDGKPLPGVRVEVRMLRHAFGWGTAVSARQLLAEGADAERYRRAVLDNFNMVVLENDLKWPQWERDRQPALAALRWLHDNGISRVRGHTLVWPGWRWLPPDLKELAADPAALRKRVLDHIRDEVSATSGLLEDWDVVNEPYTNHDLLDILGREEMVAWYQAAREFDPKPVLYLNDFNIIEAGGRDEKHRQHFYETIRFLLERGAPLGGIGIQGHFREPTPPEKILEILDLFSEFNLPIRITEFDFETKDEQLQAEFTRDFLTLCFSHPRVDAFLMWGFWEGRHWRPDGAMLRKDWSEKPVYRVWRELVHGRWRTEAEGVTGDDGSFRLRAFRGEYEIRAGGRTLRAACPSVVRVRLE

*>* Tc15-Xyn10

MALAQSGPPLRALAEKRNFQIGAAVEPSLLLQEPEYARILAQEFNLVVAENVMKWGALQTSRGQYNFALADLLMDFAQKNRMAVRGHTLVWHQQLPRWMYGGFSPAEMEAILREHIQTVVGRYRGRIAYWDVVNEAVGDDAKLRPTPFEVLPDYLEKAFRLAREADPQAKLFYNDYGAEGLGPKSDAIYALLKGLKERGVPLDGVGFQVHVDLGFSPGAVRMAENLERFARLGLEIHITEMDVRLGGPGSRAERLEKQAQVYREVMRVCLGQPRCKAFTLWGFTDAHSWRSASEPLIFDADYKPKPAYLALQQALQRP-
